# Analysis of Bacterial and Archaeal Communities along a High-Molecular-Weight Polyacrylamide Transportation Pipeline System in an Oil Field

**DOI:** 10.3390/ijms16047445

**Published:** 2015-04-02

**Authors:** Cai-Yun Li, Jing-Yan Li, Serge Maurice Mbadinga, Jin-Feng Liu, Ji-Dong Gu, Bo-Zhong Mu

**Affiliations:** 1 State Key Laboratory of Bioreactor Engineering and Institute of Applied Chemistry, East China University of Science and Technology,130 Meilong Road, Shanghai 200237, China; E-Mails: licaiyun@mail.ecust.edu.cn (C.-Y.L.); smmbadinga@ecust.edu.cn (S.M.M.); ljf@ecust.edu.cn (J.-F.L.); 2 Daqing Oilfield Limited Company, Daqing 163453, China; E-Mail: ljingyan@petrochina.com.cn; 3 School of Biological Sciences, The University of Hong Kong, Pokfulam Road, Hong Kong, China; E-Mail: jdgu@hkucc.hku.hk

**Keywords:** microbial community, 16S rRNA gene, CCA, oilfield, HPAM degradation

## Abstract

Viscosity loss of high-molecular-weight partially hydrolyzed polyacrylamide (HPAM) solution was observed in a water injection pipeline before being injected into subterranean oil wells. In order to investigate the possible involvement of microorganisms in HPAM viscosity loss, both bacterial and archaeal community compositions of four samples collected from different points of the transportation pipeline were analyzed using PCR-amplification of the 16S rRNA gene and clone library construction method together with the analysis of physicochemical properties of HPAM solution and environmental factors. Further, the relationship between environmental factors and HPAM properties with microorganisms were delineated by canonical correspondence analysis (CCA). Diverse bacterial and archaeal groups were detected in the four samples. The microbial community of initial solution S1 gathered from the make-up tank is similar to solution S2 gathered from the first filter, and that of solution S3 obtained between the first and the second filter is similar to that of solution S4 obtained between the second filter and the injection well. Members of the genus *Acinetobacter* sp. were detected with high abundance in S3 and S4 in which HPAM viscosity was considerably reduced, suggesting that they likely played a considerable role in HPAM viscosity loss. This study presents information on microbial community diversity in the HPAM transportation pipeline and the possible involvement of microorganisms in HPAM viscosity loss and biodegradation. The results will help to understand the microbial community contribution made to viscosity change and are beneficial for providing information for microbial control in oil fields.

## 1. Introduction

Partially hydrolyzed polyacrylamide with high-molecular-weight has been employed extensively as an oil-displacing agent for enhanced oil recovery in the petroleum industry [1,2]. A viscosity loss of the HPAM solution occurred in a consecutive transportation pipeline of a water injection system after six years operation. Subsequently this viscosity loss caused a decrease in oil recovery. The factors contributing to viscosity loss of the HPAM solution are speculated to be pH, temperature, salinity, mechanical force, shear force and microbial degradation. With the exception of microbial degradation [3], the other factors are controllable and have been studied thoroughly [4,5,6]. It has been reported that microorganisms are capable of metabolizing polymers and low-molecular-weight polymerization products [7], and bacteria such as *Bacillus sphaericus*, *Acinetobacter* sp., *Bacillus cereus* and *Bacillus* sp. isolated from soils and oilfields have been directly implicated in the biodegradation of HPAM by hydrolyzing the amide chain or cleaving the carbon chain to reduce HPAM viscosity [8].

With the advantage of the development of deoxyribonucleic acid (DNA)-based molecular techniques, microbial communities may be characterized more accurately and many microorganisms including culturable and non-culturable ones were identified in environments like soil [9], seawater [10], inland water [11] and oilfields [12,13,14]. Recently, the microbial communities in oilfields both of high and low temperatures have been analyzed [15,16,17]. The microbial communities related to the viscosity loss of the HPAM solution need to be analyzed.

In this study, the bacterial and archaeal compositions in different samples along the transportation pipeline were analyzed employing PCR (polymerase chain reaction)-amplification of 16S rRNA (ribonucleic acid) gene and clone library construction method. The corresponding relationship between environmental conditions and HPAM properties with microbial communities were revealed by CCA.

## 2. Results and Discussion

**Figure 1:**
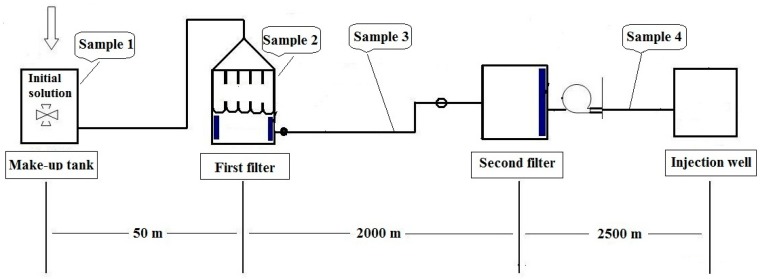
Schematic diagram showing the water injection system and the sampling sites.

In this work, four samples were collected. They are the initial solution of sample 1 (S1) collected from make-up tank; Sample 2 solution (S2) gathered from the first filter; Sample 3 solution (S3) obtained between the first and the second filter; Sample 4 solution (S4) obtained between the second filter and the injection well, respectively. The diagram of the water injection system and sampling sites are shown in Figure 1.

### 2.1. Physicochemical Information of Samples

Environmental factors and HPAM physicochemical properties of the samples were measured using different chemical methods (Table 1).

**Table 1:** Environmental factors and HPAM physicochemical properties of samples.

Samples	S1	S2	S3	S4
Temperature (°C)	25	24	23	24
Sampling distance (m)	0	50	1500	2050
pH	6.68	7.50	7.52	7.59
Solids content (%)	0.451	0.596	0.554	0.469
Viscosity (mPa·S)	1320	1071	1056	865
Na^+^ (mg·L^−1^)	805.0	689.0	466.0	442.0
K^+^ (mg·L^−1^)	38.1	26.2	31.1	6.1
Ca^2+^ (mg·L^−1^)	43.5	23.1	46.3	64.0
Mg^2+^ (mg·L^−1^)	11.0	15.9	9.1	14.0
Cl^−^ (mg·L^−1^)	709.0	144.2	473.0	173.0
NH_4_^+^ (mg·L^−1^)	160.5	60.4	95.0	62.0
NO_3_^−^ (mg·L^−1^)	11.0	15.4	48.9	9.0
SO_4_^2−^ (mg L^−1^)	561.3	298.5	190.0	58.7
PO_4_^3^^−^ (mg·L^−1^)	Nd	Nd	Nd	Nd
Formate (mg·L^−1^)	Nd	0.16	Nd	Nd
Acetate (mg·L^−1^)	0.14	2.82	3.68	2.15
Propionate (mg·L^−1^)	Nd	0.10	Nd	Nd
Butyrate (mg·L^−1^)	0.01	0.17	Nd	Nd
H_2_ (mmol·L^−1^)	Nd	0.06	Nd	Nd
CH_4_ (mmol·L^−1^)	Nd	1.36	0.92	0.05
CO_2_ (mmol·L^−1^)	Nd	32.35	4.14	7.97
HPAM concentration (mg·L^−1^)	5240	4280	4010	2940
HPAM hydrolysis degree (%)	32.55	35.07	39.65	41.13
HPAM *M*_η_ (×10^6^)	17	6.24	5.49	2.12
HPAM *R*_h_ (nm)	2394.8	692.5	573.5	282.1
HPAM PDI	0.363	0.318	0.312	0.436

(Nd: undetected; *M*_η_: viscosity-average molecular weight; *R*_h_: hydrodynamic radius; PDI: Polydispersity index).

HPAM properties of viscosity, concentration, *M*_η_ and *R*_h_ were decreasing, but the hydrolysis degree and pH are increasing with the sampling distance from S1 to S4. Laser light scattering testing (LLS) results of *R*_h_ and PDI are shown in Figure S1. *R*_h_ results indicated that the particle size of HPAM in the samples were reduced, which partially come from *M*_W_ (molecular weight) changing especially for the lowest *R*_h_ ones and lower *M*_W_ of HPAM can indicate HPAM degradation. Volatile fatty acids (formate, acetate, propionate and butyrate) were measured and high content of acetate were found in S2, S3 and S4 than in S1. The results indicated that the volatile fatty acids were mainly produced in this system. The detection of H_2_, CH_4_ and CO_2_ indicates the possibility of methanogens and sulfate-reducing bacteria (SRB) existing in the community. In addition, unpleasant smell, characteristic of H_2_S, occurs in the samples of S2, S3 and S4 when sampling.

### 2.2. Bacterial and Archaeal Community Compositions

The obtained 16S rRNA gene sequences belong to different operational taxonomic units (OTUs) and all of the valid sequences and OTUs were counted. The coverage and Shannon-Wiener index were calculated as shown in Table 2. Bacterial and archaeal community compositions were analyzed using phylogenetic analyses of 16S rRNA gene clone library [18].

**Table 2:** 16S rRNA gene and clone library analysis information of samples.

Samples	S1	S2	S3	S4
**Bacteria**				
Valid sequences	159	70	80	121
OTU	15	26	12	25
Coverage	0.9000	0.8143	0.9000	0.8843
Shannon-Wiener index (nats)	0.8035	2.8780	1.5009	2.3415
**Archaea**				
Valid sequences	30	78	102	29
OTU	14	6	6	6
Coverage	0.7000	0.9615	0.9608	0.8966
Shannon-Wiener index	2.2465	1.0857	0.4670	1.3863

**Figure 2:**
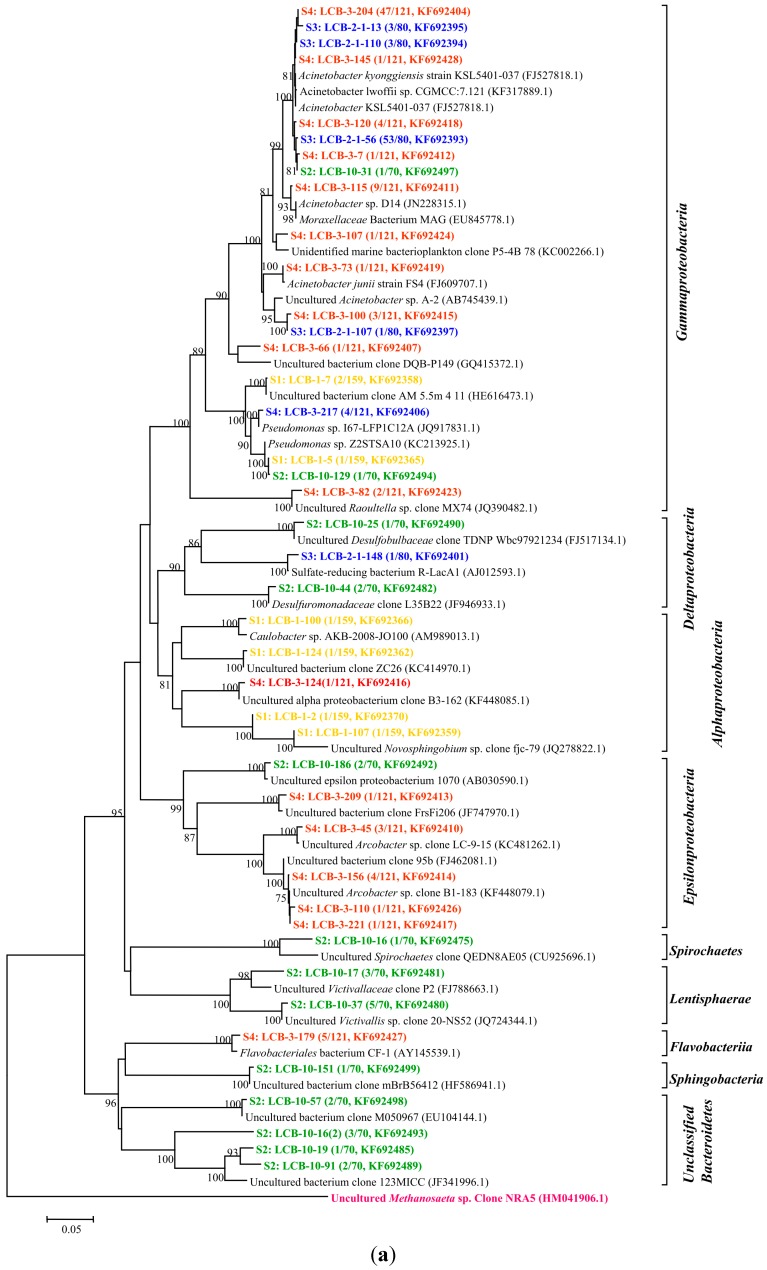

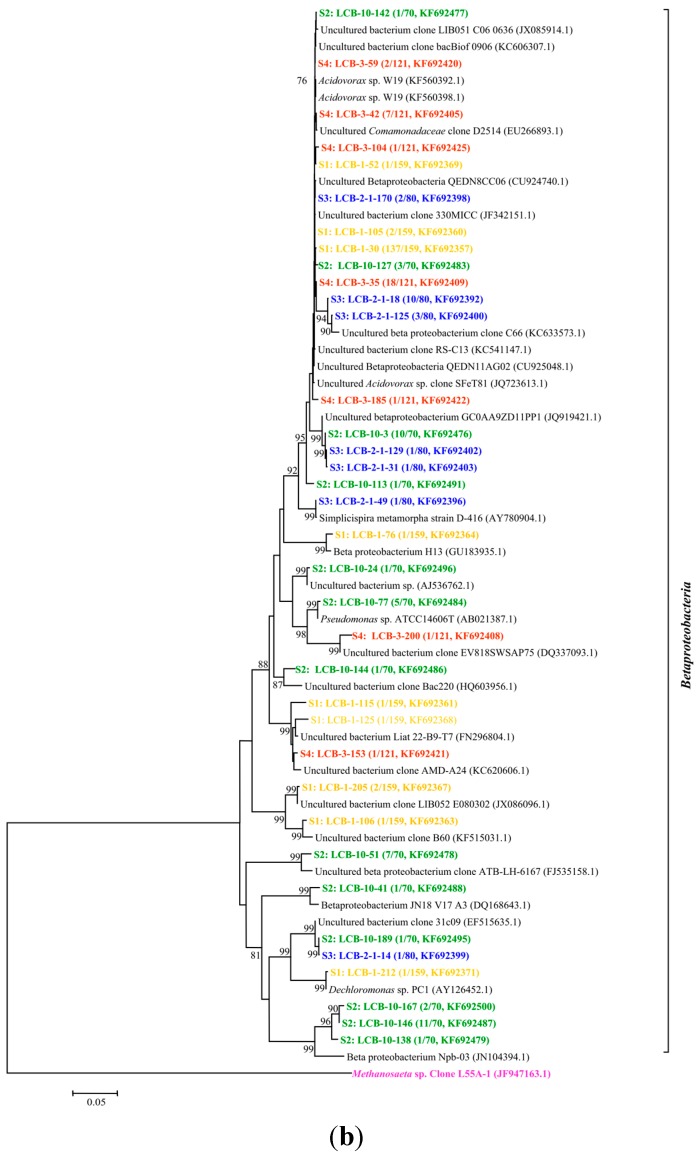
(**a**) Phylogenetic tree of bacterial 16S rRNA gene clones retrieved from four samples showing the distributions of OTUs and closely related sequences from GenBank database. The bootstrap values at the nodes of ≥75% (*n* = 1000 replicates) are reported. The scale bar represents 5% sequence divergence. The numbers in parentheses indicate the frequencies of appearance of identical clones in the total analyzed clones followed the accession number in GenBank. *Methanosaeta* sp. clone was used as outgroup; (**b**) Phylogenetic tree of bacterial 16S rRNA gene clones retrieved from samples showing the distributions of OTUs and closely related sequences from GenBank database. The bootstrap values at the nodes of ≥75% (*n* = 1000 replicates) are reported. The scale bar represents 5% sequences divergence. The numbers in parentheses indicate the frequencies of appearance of identical clones in the total analyzed clones followed the accession number in GenBank. The *Methanosaeta* sp. clone was used as outgroup. The gene sequences from different samples are assigned with different colors: S1 in yellow, S2 in green, S3 in blue and S4 in red.

All of the gene sequences are affiliated with the members of the phyla Proteobacteria, Spirochaetes, Lentisphaerae and Bacteroidetes and the classes of Betaproteobacteria, Gammaproteobacteria, Epsilonproteobacteria, Deltaproteobacteria, Alphaproteobacteria, Spirochaetes, Lentisphaeria, Sphingobacteria and Flavobacteriia. The results are presented in two phylogenetic trees (Figure 2a,b); (note: The microorganisms in sample S1 are represented by the microbial communities in the tap water for dissolving HPAM). The gene sequences from different samples are assigned with different colors: S1 in yellow, S2 in green, S3 in blue and S4 in red.

**Figure 3:**
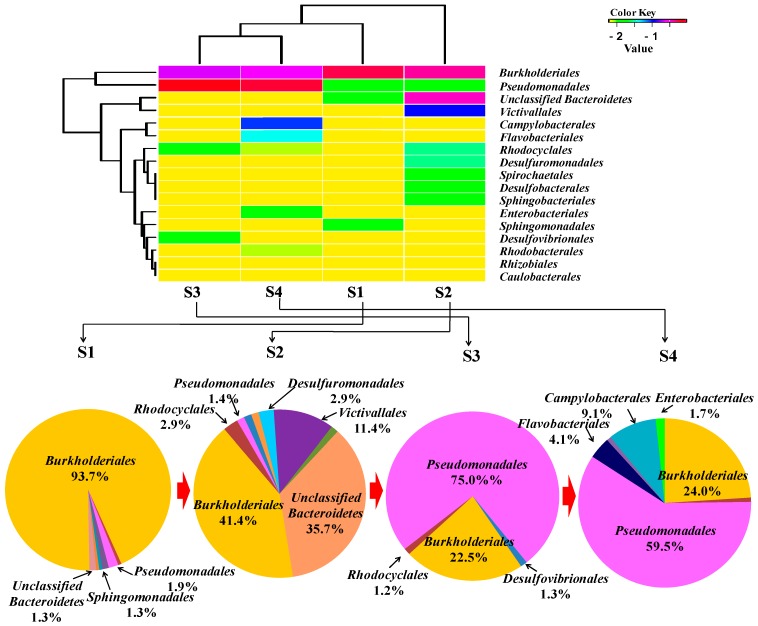
Relative abundance of bacterial orders detected in samples of an oilfield pipeline.

The relative abundances of bacterial orders were determined and are shown in Figure 3.

The relative abundances of bacterial orders were determined and are shown in Figure 3.

Along the transportation pipeline, the bacterial community compositions from sample 1 (S1) to sample 4 (S4) changed. *Burkholderiales* and *Pseudomonadales* are the main dominating orders compared with the other community. However, the *Burkholderiales* decreased in abundance from 94% to 24%, with increasing distance from the make-up tank, whereas the *Pseudomonadales* increased in relative abundance from 2% to 60%. *Rhodocyclales* remained at low abundance from 0.6% to 0.8% with a bit of an increase to 2.9% and 1.3% in S2 and S3, respectively. Sulfate-reducing bacteria of *Desulfobacterales* (1.4%), *Desulfuromonadales* (2.9%) and *Desulfovibrionales* (1.3%) were detected in S2 and S3, respectively. *Campylobacterales* (9.1%), *Enterobacteriales* (1.7%) and *Flavobacteriales* (4.1%) were only shown in S4. The heat map was drawn [19] and is also shown in Figure 3, which displays that the bacterial community compositions of S1 are close to those of S2 and those of S3 are close to S4. From *Burkholderiales* to *Caulobacterales*, the abundance of each order is decreasing following the sequence from top to bottom.

Through the analysis of HPAM physicochemical properties, it was found that the viscosity of HPAM gradually decreased along the transportation pipeline, especially from S3 to S4. As some bacteria are heterotrophs, they can utilize carbohydrates as sources. HPAM also can be used as N, C or C/N sources in this environment. Some isolates were reported of being responsible for HPAM biodegradation directly, but unfortunately their sequences information is not available in the GenBank database. For example, *Enterobacter agglomerans* utilized HPAM as sole carbon or nitrogen source and degraded high *M*_W_ HPAM to low *M*_W_ ones [20]. The same order of *Enterobacteriales* was found in S4. *Pseudomonas migula* CJ419 was found to degrade HPAM viscosity by 30.4% [21], and bacteria belonging to *Pseudomonadales* sp. occurred with high abundance in both S3 and S4. SRB of *Clostridium bifermentans* H1 was found to utilize HPAM as sole carbon source, hydrolyze the amide group, degrade the side chain and change some functional groups, which finally results in reducing HPAM viscosity [22]. SRB also reduced the viscosity of 1000 mg/L HPAM solution by 20% after seven days of cultivation at 30 °C [23]. Similarly, SRB were detected in S2 and S3 including *Desulfovibrionales* in S3, *Desulfobacterales* and *Desulfuromonadales* in S2. The SRB metabolic product of sulfide was proved to reduce HPAM viscosity as well [24]. *Acinetobacter* sp. can degrade HPAM reducing its molecular weight and lowering viscosity by 78% [8]. These bacteria were detected in both S3 and S4 with high appearance frequency. HPAM viscosity reduced significantly from 1056 to 865 mPa·S, and from S3 to S4, and *Acinetobacter* sp. occurred with high abundance indicating that these bacteria have a large potential to be the main microorganisms to reduce HPAM viscosity.

Although isolates such as *Bacillus cereus* HWBI, *Bacillus flexu* HWBII, *Bacillus cereus* PM-2 and *Bacillus* sp. PM-3 were described to utilize polyacrylamide such as nitrogen and carbon sources [3,25], they were not detected in the analysis results of four samples. For other bacteria detected in the samples, their possible participation in HPAM biodegradation is not known. *Sphingobacteriales* are a main member of the *Bacteroidetes* and its principal genus is *Cytophaga*, which can degrade cellulose [26]. *Victivallales* is an uncultured bacterium, and was detected in bovine rumen, which has an auxiliary function on vegetal fiber degradation [27]. *Flavobacterials* can be used to degrade a mixture of anthracene, phenanthrene and pyrene [28]. Thus, these groups may possibly participate in HPAM biodegradation.

**Figure 4:**
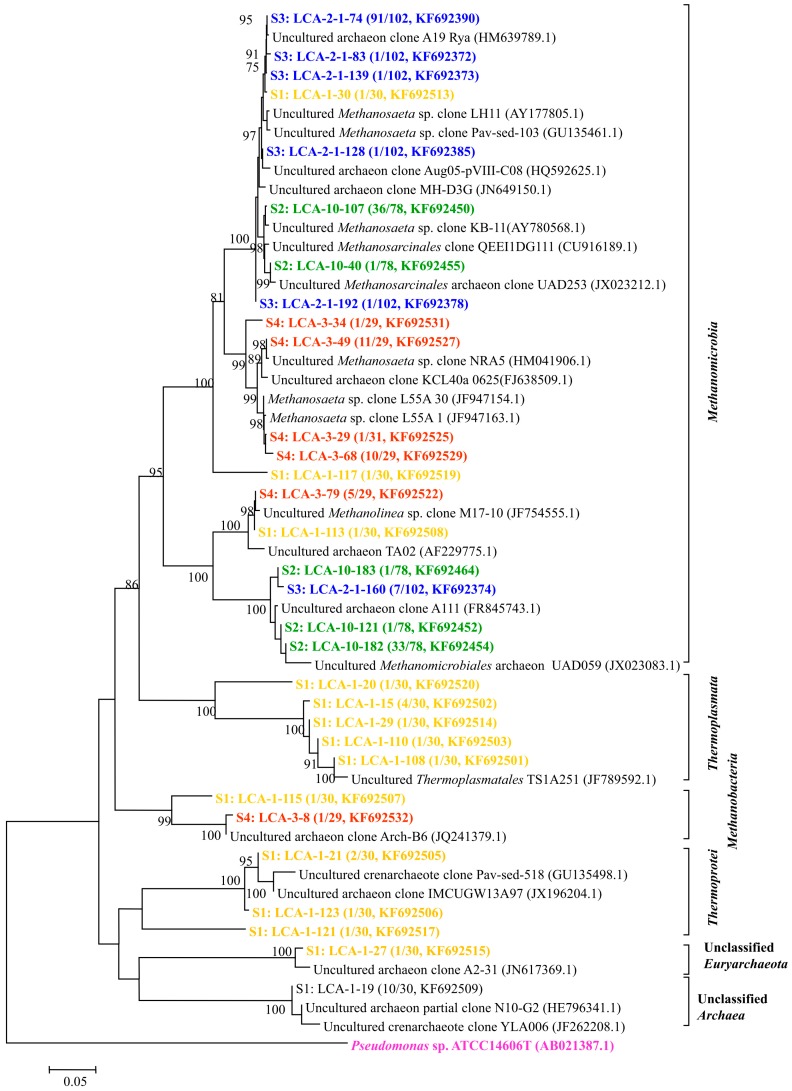
Phylogenetic tree of archaeal clones from the samples of S1–S4 showing the distributions of OTUs and closely related sequences from GenBank database. The bootstrap values at the nodes of ≥75% (*n* = 1000 replicates) are reported. The scale bar represents 5% sequences divergence. The numbers in parentheses indicate the frequencies of appearance of identical clones in the total analyzed clones followed with the accession number. *Pseudomonas* sp. clone ATCC14606 was used as outgroup.

Archaea are essential and occurred together with bacteria. Archaeal community compositions were analyzed employing the same steps as the above bacterial community analysis and four specific groups were detected, as shown in Figure 4.

**Figure 5:**
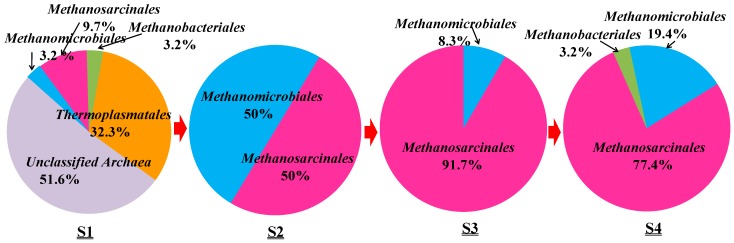
Relative abundance of archaeal orders detected in S1–S4 samples from an oilfield pipeline.

Based on the phylogenetic analysis, the relative abundance of archaeal orders in the samples from S1 to S4 was determined and is shown in Figure 5.

Along the transportation pipeline, *Methanomicrobiales* and *Methanosarcinales* are the two main orders occurring in the samples from S2 to S4. *Methanosarcinales* occurred in S2 with the relative abundance of 50%, in S3 with 91.7% and finally in S4 with 77.4% abundance. *Methanomicrobiales* occurred in S2 with 50% abundance but in S4 with only 19.4%. *Methanobacteriales* were detected twice with 3.2% abundance, respectively. The heat map also showed that archaeal communities of S3 are close to those of S4 (Figure S2).

Archaea were generally found to thrive in harsh environments, but they are also found in soil, ocean, marshland and oilfields [29]. Three orders of archaea were detected in all samples including *Methanobacteriales*, *Methanomicrobiales* and *Methanosarcinales*, and all of them are methanogens. *Methanobacteriales* and *Methanomicrobiales* are classified to be hydrogenotrophic methanogens utilizing H_2_ and CO_2_ as substrate to generate methane [30]. *Methanosarcinales* are classified to be acetoclastic methanogen utilizing acetate to generate methane [15]. Since acetate, H_2_, CO_2_ and CH_4_ were separately found in four samples, only acetate comes from the tap water and some of them were supposed to be related with HPAM biodegradation. It is assumed that these archaea utilized acetate or, H_2_/CO_2_ to produce CH_4_. Methanogenesis can then accelerate HPAM biodegradation indirectly [31].

### 2.3. Statistical Analysis of Canonical Correspondence Analysis (CCA)

**Figure 6:**
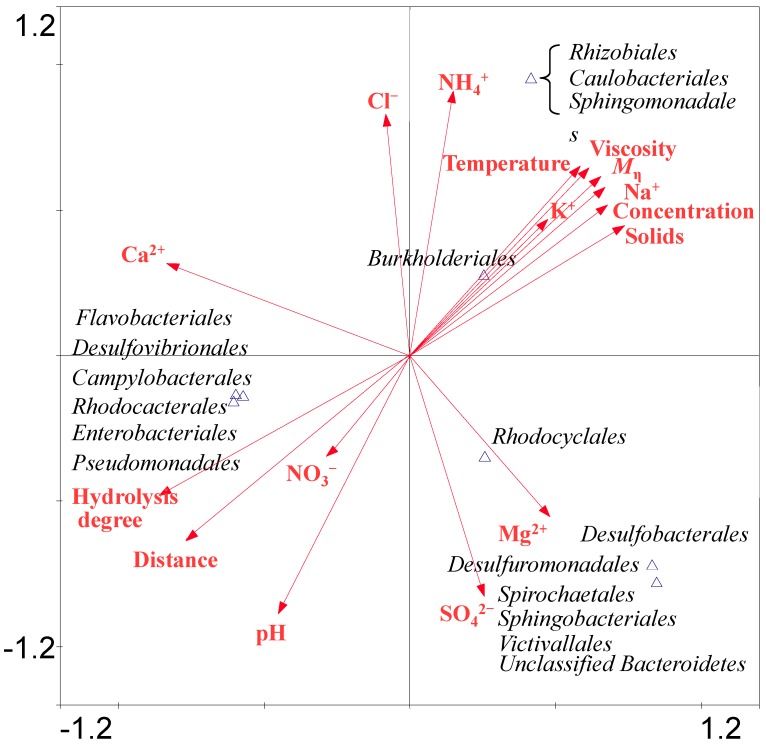
CCA ordination plots for the two dimensions to show the relationship between the bacterial diversity and environmental parameters analyzed using a 16S rRNA gene sequences in the water injection system of four samples. Correlations between environmental variables and CCA axes are represented by the length and angle of arrows (environmental factor vectors). Triangle means one or several species appearing position.

The correlationship between the distribution of bacterial or archaeal species and environmental factors is revealed by CCA analysis (Figure 6).

Bacterial CCA analysis revealed that the bacteria belonging to *Burkholderiales*, *Sphingomonadales*, *Rhizobiales* and *Caulobacterales* were positively correlated with temperature, *M*_η_, hydrolysis degree, HPAM concentration and viscosity, the concentration of Na^+^, K^+^ and NH_4_^+^. The bacteria belonging to *Sphingobacteriales*, *Victivallales*, *Desulfuromonadales*, *Desulfobacterales*, *Spirochaetales* and *Rhodocyclales* were positively correlated with the concentration of Mg^2+^ and SO_4_^2−^. The members of the *Enterobacteriales*, *Rhodobacterales*, *Desulfovibrionales*, *Flavobacteriales*, *Campylobacterales* and *Pseudomonadales* were positively correlated with NO_3_^−^ concentration, pH, hydrolysis degree and distance. As abundant anionic and cationic ions were detected in the samples, the SO_4_^2−^ can be utilized by *Desulforomonadales* in S2. HPAM contains many amide groups, which can be transformed into NH_4_^+^ and then NH_4_^+^ can be oxidized to NO_3_^−^ by bacteria. *Desulfovibrionales* have a nitrate reductase to reduce NO_3_^−^ into NH_4_^+^ [32]. Archaeal CCA results (Figure S3), suggesting that archaea belonging to *Methanosarcinales* were positively related with pH, hydrolysis degree and NO_3_^−^ concentration and sampling distance. *Methanomicrobiales* were positively related to Mg^2+^ and SO_4_^2−^ concentration. *Methanobacteriales* was positively related to Cl^−^ and NH_4_^+^ concentration. *Thermoplasmatales* was positively related to *M*_η_, viscosity, solids content and concentrations of K^+^ and Na^+^.

The above analysis of the samples indicates that the microbial community diversity has a positive correlation with HPAM physicochemical properties. It is likely that the bacteria detected in the analyzed samples were involved in reducing HPAM viscosity. It is also likely that abundant members of the genus *Acinetobacter* were potentially involved in HPAM biodegradation which is consistent with the large HPAM viscosity loss from S3 to S4. Implication of other groups of microorganisms in HPAM biodegradation is not excluded.

## 3. Experimental Section

### 3.1. Description of Sampling

Four samples containing HPAM (Daqing Huagong Chemical Company, Daqing, China) were taken from four different sites of the water injection system in a Daqing oil field. In this system, potable water is used to dissolve polyacrylamide to be a solution. Sample 1 through sample 4 were designated as: S1 for initial solution containing approximately 5000 ppm of HPAM; S2 for HPAM solution prior to the first filter; S3 for HPAM solution between the first and the second filter; S4 for HPAM solution between the second filter and the injection well (Figure 1). All four samples were initially prepared with potable water [33]. Approximately ten liters of samples were collected directly into sterile containers together with the water. The containers were tightly sealed and immediately transported back to the laboratory for analysis.

### 3.2. Sample Analysis

Solid content and viscosity were tested according to the standard method SY/T5862-93 [34]. Hydrolysis content and *M*_η_(viscosity-average molecular weight) were analyzed based on the standard method GB 17514-2008 [35,36]. The content of HPAM referring as the total amount of amide-N was determined by the starch-cadmium iodide colorimetric method [37]. The concentration of formate, acetate, propionate, butyrate, anionic ions and cationic ions in samples were quantified using Dionex 600 ion chromatography (Triad Scientific, Inc., Manasquan, NJ, USA) using DIS-5C and DIS-5A suppressors. Anions were separated on an AS11 HC analytical column (4 × 250 mm) with an AG11 HC guard column (4 × 50 mm), (both columns, Dionex, Manasquan, NJ, USA). Cations were separated on a CS 12A analytical column (4 × 250 mm) with a CG 12A guard column (4 × 50 mm) [38]. Samples were measured by a laser light scattering (LLS) spectrometer (ALV/CGS-2022) equipped with an ALV-High QE APD detector and an ALV-5000 digital correlator using a He-Ne laser (wavelength λ = 632.8 nm). The Laplace inversion of each measured intensity-intensity time correlation function *G*(2) (*t*,*q*) is in self-beating mode, which can result in a line width distribution *G*(Γ). The translational diffusion coefficient *D* is calculated by the slope of the Γ *vs.* *q*^2^ plot (Γ is decay time). Then the hydrodynamic radius < *R*_h_ > is obtained by the Stokes-Einstein equation *R*_h_ = *k*_B_*T*/(6πη*D*), (*k*_B_, *T* and η are the Boltzmann constant, temperature, and the solvent viscosity, respectively). Polydispersity index (PDI) was determined at the scattering angle of 90° [39]. The headspace gas like CH_4_, H_2_ and CO_2_ in the bottles were analyzed by gas chromatography (GC) equipped with a thermal conductivity detector (TCD) to detect H_2_ and a flame ionization detector (FID) for CH_4_. CO_2_ which is converted into CH_4_ by a conversion furnace (GC112A, Shanghai Precision Scientific Instrument Co., Ltd., Shanghai, China) [40].

### 3.3. Nucleic Acid Extraction and PCR Amplification

The samples were first filtered through polycarbonate membrane filters (0.22-µm-pore-size, Millipore, Bedford, MA, USA) aseptically in the laboratory. Afterwards the membranes were put into a sterile centrifuge tube subjected to beads beating. Total genomic community DNA was extracted by using AxyPrep™ Bacterial Genomic DNA Maxiprep Kit (Axygen Biosciences, Inc., Santa Clara, CA, USA) and stored at −20 °C before testing [41,42]. Agarose gel electrophoresis (1.0%, *w*/*v*) was used to determine the yield and integrity of DNA extraction. Universal PCR primer sets (Table 3) were applied for amplification of bacterial and archaeal 16S rRNA gene sequences, respectively [43].

**Table 3:** Primer sets information.

Primer Set	Target Organisms	Sequences (5'–3')	Annealing Temperature
8F	Bacteria	AGAGTTTGATYMTGGCTCAG	52 °C
805R	Bacteria	GACTACCAGGGTATCTAATCC	52 °C
A109F	Archaea	ACKGCTCAGTAACACGT	60 °C
A1041R	Archaea	GGCCATGCACCWCCTCTC	60 °C

The reaction mixture for PCR amplification contained 9 µL sterile ultrapure water, 2× Taq PCR Master Mix (Tiangen Biotech Co., Ltd., Beijing, China), 1 µL of the forward and reverse primers in one primer set (GenScript Co., Ltd., Nanjing, China), and 2 µL DNA template (80 ng DNA templates [44]). PCR amplification reaction was performed under the following conditions: initial denaturation of DNA at 94 °C for 1 min, followed by 30 cycles of 1 min denaturation at 94 °C, primer annealing at 52 °C (for bacteria)/60 °C (for archaea) for 1 min and extension at 72 °C for 1 min, and a final extension step at 72 °C for 10 min.

### 3.4. 16S rRNA Gene Clone Sequencing and Accession Numbers

The PCR products were gel-purified (U-Gene Gel Extraction kit II, Shanghai, China). Purified bacterial and archaeal DNAs were ligated with pMD^®^19-T simple vector (Takara Biomedical Technology, Dalian, China) by adding 4.5 µL of DNA and 0.5 µL of pMD^®^ 19-T Simple vector to a 200 µL centrifugal tubes for reaction at 4 °C overnight. The ligated DNAs were then transformed into the competent *Escherichia coli* cells according to the manufacturer’s instruction (Shanghai Lifefeng Biotech Co., Ltd., Shanghai, China). The inserted 16S rRNA gene fragments were screened by PCR amplification with the vector specific primer set of M13-47 (5'-CGCCAGGGTTTTCCCAGTCACGAC-3') and RV-M (5'-GAGCGGATAACAATTTCACACAGG-3'). The reactions were carried out under the following conditions: Initial DNA denaturation at 95 °C for 5 min, 20 cycles of 1 min denaturation at 94 °C, primer annealing at 52 °C for 1 min, and extension at 72 °C for 1 min, a final extension at 72 °C for 10 min. The positive clones in each library were selected for sequencing with an automated ABI Prism 377 analyzer by using M13-47 sequencing primer. Primers and vectors were manually removed from the obtained DNA sequences using MEGA 5.0 software [45]. Chimeric sequences were verified and removed by using Bellerophon [46]. Sequences were clustered into the same operational taxonomic units (OTUs) using FastGroup II at a 97% similarity [47]. One representative sequence was chosen from each OTU to compare with the submitted sequences using the BLASTN network service [48]. The sequence without chimeras was initially submitted to the BLASTN program to determine their closest phylogenetic relatives. Phylogenetic trees were constructed based on the neighbor-joining algorithm using MEGA software. The confidence levels to the nodes of the consensus trees were assigned using bootstrap analysis with 1000 replicates. Partial 16S rRNA gene sequences of bacterial and archaeal genes were deposited in the GenBank database with the accession numbers from KF692357 to KF692428 and from KF692450 to KF692532, respectively.

## 4. Conclusions

Four samples were collected along a transportation pipeline system for high-molecular-weight polyacrylamide solution to be injected in an oil field. Microbial community compositions and the physicochemical properties of the samples were analyzed. Diverse bacterial groups were detected including Betaproteobacteria, Epsilonproteobacteria, Gammaproteobacteria, Alphaproteobacteria, Deltaproteobacteria, Spirochaetes, Lentisphaeria, Sphingobacteria and Flavobacteria together with the archaeal members of the orders *Methanomicrobiales*, *Methanosarcinales*, *Methanobacteriales* and *Thermoplasmatales*. The microbial community of S1 was similar to the one of S2 and that of S3 was similar to S4. Bacteria and archaea exhibited a positive correlationship with different HPAM physicochemical properties and environmental parameters. Our data, in conjunction with other literature reports, suggest that members of the genus *Acinetobacter* detected with high abundance in samples S3 and S4 would have been mainly involved in HPAM viscosity loss. This work will help to understand the microbial community contribution to viscosity change in this system and are beneficial for providing information for microbial control in oil fields.

## Supplementary Materials

Supplementary materials can be found at http://www.mdpi.com/1422-0067/16/04/7445/s1.

## Abbreviations

HPAM: hydrolyzed polyacrylamide
CCA: canonical correspondence analysis
LLS: laser light scattering
*M*_W_: molecular weight
*M*_η_: viscosity-average molecular weight
*R*_h_: hydrodynamic radius
PCR: polymerase chain reaction
RNA: ribonucleic acid
GC: gas chromatography
TCD: thermal conductivity detector
FID: flame ionization detector
DNA: deoxyribonucleic acid
OTUs: operational taxonomic units
PDI: polydispersity index
S1: sample 1
S2: sample 2
S3: sample 3
S4: sample 4
