# Supplementary material for: Analysis of Bacterial and Archaeal Communities along a High-Molecular-Weight Polyacrylamide Transportation Pipeline System in an Oil Field
Source: Int J Mol Sci. 2015 Apr 2;16(4):7445–61. doi: 10.3390/ijms16047445 (PMC4425027; doi:10.3390/ijms16047445)
Supplement: Supplementary file 1 [file ijms-16-07445-s001.pdf]

Supplementary Information

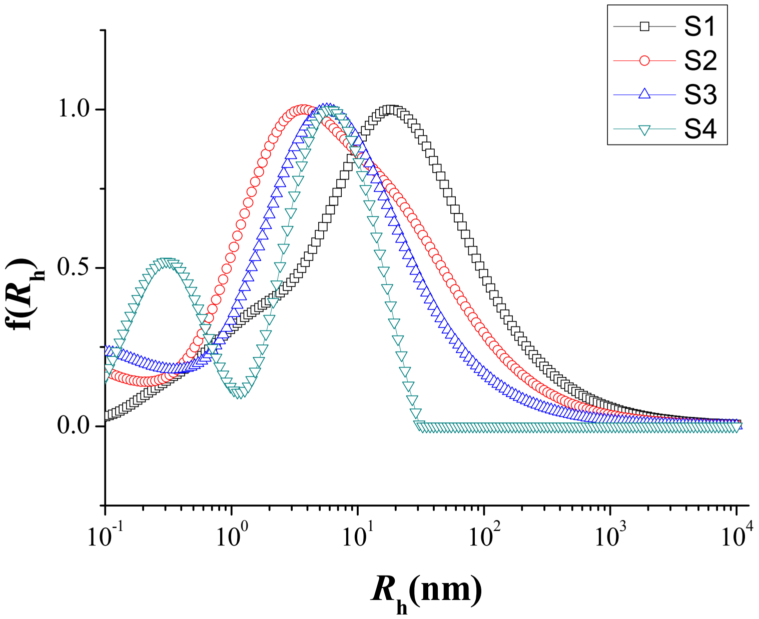

Figure S1. A typical hydrodynamic radius distribution $f\,(R_h)$ of the particles for the four samples. The scattering angle is 90°.

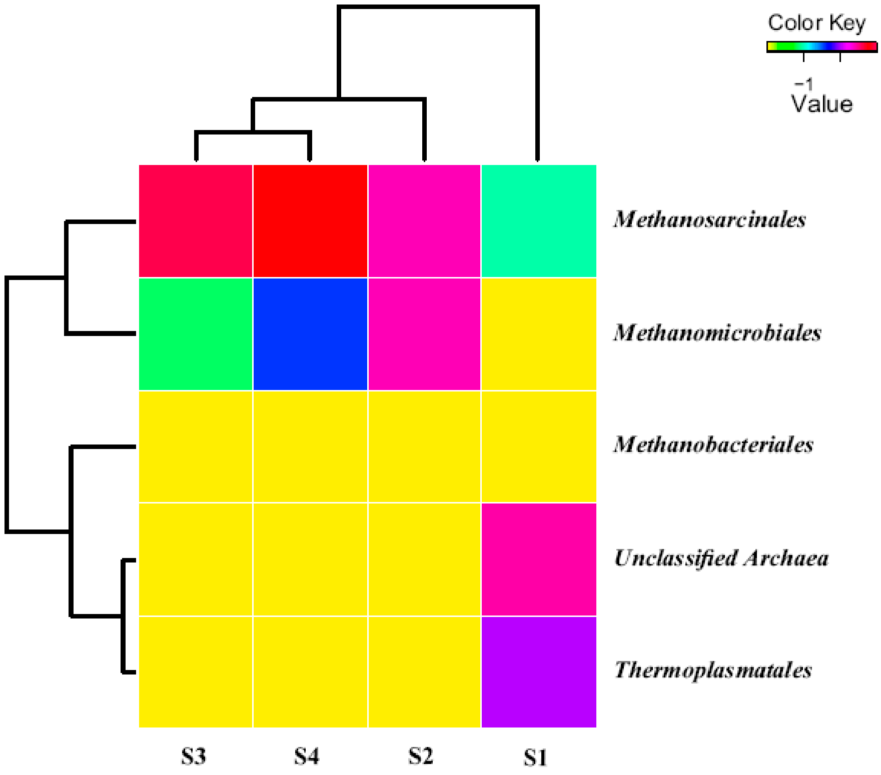

Figure S2. Archaeal heat map of the 4 samples.

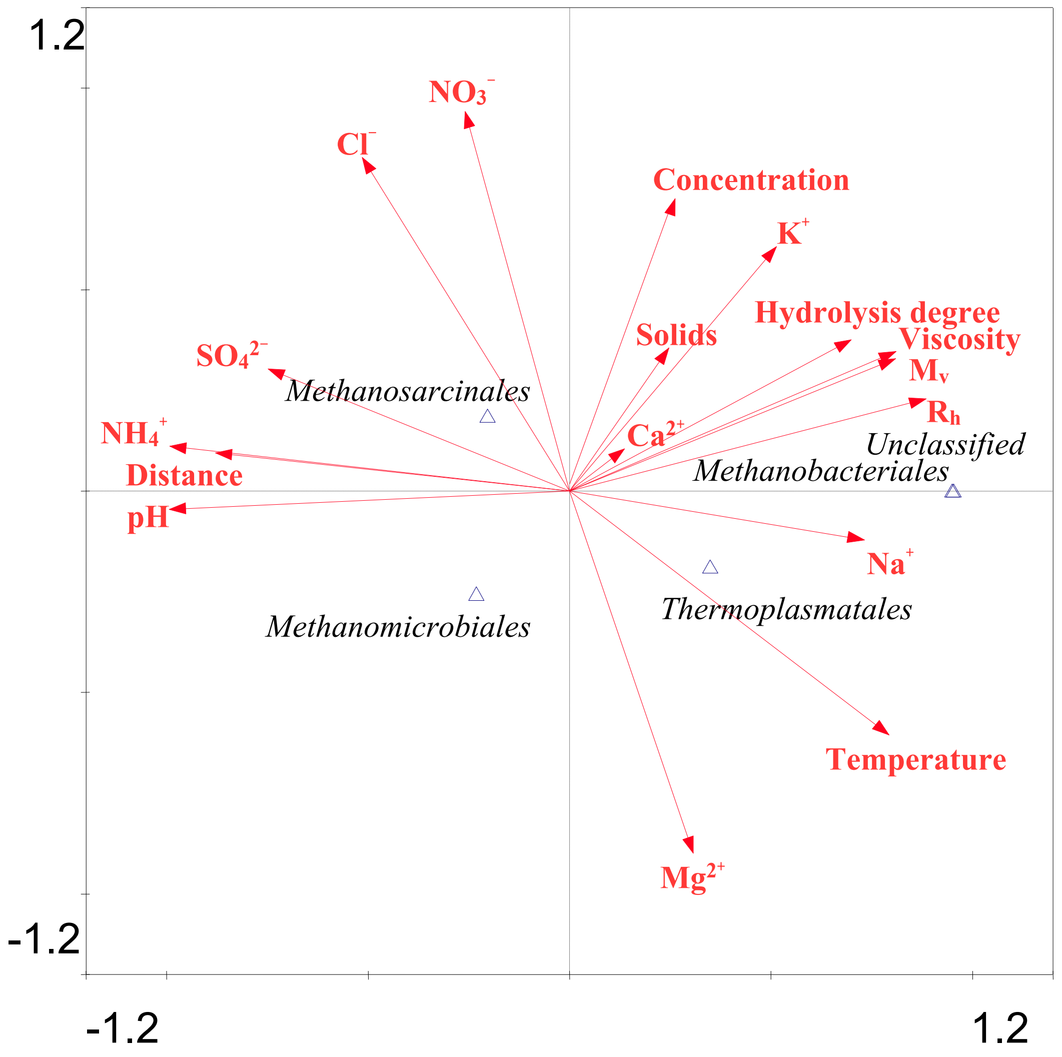

Figure S3. Canonical correspondence analysis (CCA) ordination plots for the two dimensions to show the relationship between archaeal diversity and environmental parameters analyzed using a 16S rRNA gene sequences in the water injection system of four samples. Correlations between environmental variables and CCA axes are represented by the length and angle of arrows (environmental factor vectors).
